# The Plea of Insanity

**Published:** 1849-04-01

**Authors:** 


### MEDICAL JURISPRUDENCE—THE PLEA OF INSANITY.

Parry v. Gardner.—This was an action of ejectment brought to recover an estate at Belcham, in the County of Hertford, said to be of the value of 10,000*l.* Sergeant Channel and Mr. Chambers appeared for the plaintiff, and Sergeant Shee and Mr. Peacock for the defendant. The case commenced on Wednesday, and occupied the court till a late hour on Friday night.

The plaintiff was Mr. Nicholas Parry, a gentleman of fortune, residing near Puckeridge, and was admitted to be the heir-at-law of a person named James Spalding, deceased, who had been the owner of the estate in question. James Spalding, during his life-time, had lived in the house with the defendant, who was a connexion of his family, and who had had the management of his estate. In 1821, James Spalding made his will, and devised the whole of his estate to the defendant; by a codicil dated in 1825, he gave other property, which he had subsequently purchased, to the defendant also. The testator died in 1847, when the defendant took possession of the estates by virtue of the will. This action was brought by the plaintiff as heir-at-law of the testator, to recover possession of the property, and the case set up by him was, that the testator, Mr. James Spalding, was not in such a state of mind as to enable him to understand the nature of a will, that he was incapable of executing such an instrument, and that the will in question had been made by him under the coercion of the defendant. In order to support this case, a great number of witnesses were examined, whose testimony showed that even while at school Mr. Spalding was treated as a boy of weak intellect; that after he left school, he resided with and was under the charge of his mother, who, when he was twenty years old, used to treat him as a child. Upon the death of his mother and brother, and when he had no longer any relation to take an interest in him, he went to reside with the defendant, who had been appointed by his sister, Mrs. John Spalding, to manage the property, and according to the evidence, he was treated for a great number of years by the defendant, not like the owner of the estate, but more like a labourer; that he was dressed in mean apparel, was never allowed to take any share in the management of the farm, and that the defendant used frequently to horsewhip him, and use other personal violence towards him. It was likewise proved that from childhood the testator had been addicted to most filthy and disgusting habits, and the groom, who used to ride out with him while he was under the care of his mother, stated that he was always looked upon as a person of weak intellect, and was treated as such by the family. Evidence was also adduced to show that he was continually in the habit of getting drunk, and that he was never seen with more money in his possession than a few pence at a time, and that he used to borrow sixpence or a shilling from the people about the neighbourhood, and a variety of other facts were proved, making out a very strong case with regard to his insanity, or at all events, incompetency to understand the nature of such a document as he was represented to have executed.

Sergeant Shee, on behalf of the defendant, produced a great many witnesses, who gave evidence to show that the testator had upon several occasions interfered in the management of his property, and that he had also, at different times, executed mortgage deeds, which had been prepared by some of the most respectable solicitors in the country, all of whom, however, it appeared, were dead, and it was urged that persons in such a position would not have sanctioned his taking such a proceeding, if he had not been perfectly competent to do so. Some letters and documents written by the testator, were also put in as a proof of his competency of mind.

At the conclusion of the evidence on Friday night, the learned judge summed up, and the jury retired at nine o'clock, to consider their verdict. At half-past ten they returned, giving a verdict for the plaintiff, thus establishing the title of the heir-at-law, and setting aside the will.

---

Sarah Grout, aged 31, was indicted for the wilful murder of James Grout, her son, by cutting him in the neck with a billhook.

There was a second indictment against the prisoner for the murder of Mary Anne Grout, another of her children, by the same means.

Mr. Ryland and Mr. Hamilton prosecuted. The prisoner was defended by Mr. T. Chambers.

Mr. Ryland, in opening the case, said that the evidence would leave no doubt that the prisoner had committed the dreadful act that was imputed to her, and the only question the jury would have to consider was, whether, when she did so, she was in such a state of mind as to make her responsible for her actions.

Emma Creek deposed that she was the wife of a labourer, and resided at West Thurrock, near a cottage occupied by the prisoner and her husband. They had three children, a boy seven years old, the deceased, James, four years old, and a little girl, Mary Anne, about two. On the morning of the 8th of August, last year, the eldest boy came into witness's cottage, and told her that his mother had murdered the two babies, and had nearly cut their heads off with a billhook. She immediately went to the house, accompanied by a man named Isaac Moss, and on going into the prisoner's bed-room, she saw the boy James lying on the ground, with his head nearly cut off, and the little girl was lying upon the bed, injured in a similar manner. Both the children were in their nightshirts. The prisoner was standing by the dead bodies with a billhook in her hand, which was covered with blood, and she repeatedly exclaimed—"I have murdered my children!" Mr. Moss took the billhook from her, and she then sat down by the bedside, and began to rub her hands and cry bitterly. Before this time the prisoner had always appeared very fond of the children, but for a month or two before August she had observed very strange ways about the prisoner, who seemed like a woman deranged in her mind.

By Mr. Chambers.—The prisoner frequently used to behave in a very extraordinary manner. Upon one occasion, she saw her putting a quantity of china into an old sack, and she said the reason she did so was, that a gentleman was coming to take it away. Upon another occasion, the prisoner told her she did not know what would become of them, for they were all going to hell; the prisoner then shook her violently, and it was with great difficulty she got her in-doors, and put her to bed. After she recovered from this paroxysm, she inquired eagerly where her children were, and wished to know if she had hurt any of them, or any one else. Witness had also frequently heard her say, that she wished she was dead, and that the only things she should grieve to leave were her two children.

By the Court.—The prisoner had three children, but at this time she seemed unconscious of that fact, and only talked about her two children.

Re-examined.—About a fortnight before this occurrence took place the prisoner told her that she had attempted to hang herself; and when witness asked her why she had done so, she said that her children were so undutiful that she did not know how to bear herself.

Isaac Moss confirmed the testimony of the last witness, with regard to the condition in which the children were found, and also said that when he took the billhook from the prisoner, and asked her how she came to commit such an act, she only rubbed her hands and said, in a low tone, "I have murdered them." The prisoner's husband was out harvesting when the affair happened.

Thomas Teal, the parish constable, deposed, that upon taking the prisoner into custody, he asked her how she came to commit such an act, and she told him that she intended to have killed her eldest boy, and then to have destroyed herself. At this time she appeared quite wild, and not to know what she was about; and they were obliged to watch her to prevent her from making away with herself.

Mr. R. B. Jordeson, a surgeon at South Ockenden, deposed that he was called in to see the bodies of the children on the 9th of August. They had both received injuries of the same character, the vertebræ at the back of the neck being divided and the spinal cord severed, and their death must have taken place instantaneously. The billhook that was shown him by the constable had no doubt occasioned the injury. The prisoner had been to his surgery for some medicine on the previous Saturday. He had known her for some years, and always considered her a person of weak mind, and on this occasion she was extremely dejected, and her appearance attracted the attention of his wife and every one who saw her. After the murder she exhibited a deplorable state of anxiety, coupled with stupidity and gloom, and he was satisfied that when she destroyed the children she was in an unsound state of mind.

Mr. Baron Parke asked the witness whether he thought that, at the time she killed the children, she was aware she was committing a crime in so doing?

The witness said, he was decidedly of opinion she was not.

The statement made by the prisoner before the magistrate was then put in. She said, "It has preyed upon my mind a long time to make away with myself. My children were undutiful, and I did not know what to do with them."

Mr. Chambers was about to address the jury for the prisoner, when

His Lordship asked them if they thought it was necessary he should do so, after the statement of the surgeon and the other evidence in the case.

The foreman of the jury said they did not consider it necessary. They had made up their minds to acquit the prisoner, on account of her being insane.

Mr. Baron Parke said, in that case they would say the prisoner was not guilty, on the ground of insanity, and she would then be taken care of, and would not be allowed to come out again until she was restored, if it should please God ever to restore her.

The same verdict was entered upon the second indictment, and the prisoner was then removed.

(*Tried before Baron Parke.*)

John Smith was indicted for the wilful murder of Eleanor Lawrence, at Collingbourn Ducis, on the 9th of August last.

Mr. Slade and Mr. Hadow were counsel for the prosecution; Mr. C. Smith defended the prisoner.

The facts of this case, as detailed in the evidence, are of a most extraordinary character, there being a total absence of all apparent motive for the commission of the crime with which the prisoner was charged. It appeared that the poor woman, Eleanor Lawrence, was a pauper, living at Collingbourn Ducis, and was about thirty-six years old. On the 9th of August last, she took down the dinners of some persons who were reaping in a field, joining the road leading from Collingbourn to Ludgershall. This was about twelve o'clock. She stayed with the reapers some little time, and then left to go back to Collingbourn. Nothing more was seen of her until about half-past two, when a Mr. Wick, who was driving along the Collingbourn-road, saw the woman lying by the side of the road, apparently insensible, and with her face and head covered with blood. As he was unable to stop his horse, which was much frightened, he drove on a short distance, and alarmed some reapers who were at work in a field. They returned with him to the spot, which was about a quarter of a mile from where they were reaping, and it was then discovered that the woman was Eleanor Lawrence. She was nearly dead, and bleeding from wounds in the head. She was carried home, where she lingered thirty-six hours, and then died. On a *post-mortem* examination being made, it was discovered that there were several slight lacerated wounds on the head, and that the skull had been fractured just over the right eye, which had depressed a portion of the bone, and was the cause of the poor woman's death. This wound, the medical men thought, must have been caused by some blunt instrument, such as a stone, or might have been produced by a kick. The evidence to connect the prisoner with the commission of the murder of the unfortunate woman was purely circumstantial, except his own statements. On the morning of the 9th of August, at about a quarter-past eleven o'clock, the prisoner called at a blacksmith's shop, at Ludgershall, which is a mile and a quarter from the spot where the murder was committed, and asked for a job of work. The prisoner remained talking with the smith about three-quarters of an hour. At that time he was dressed in a fustian jacket and trowsers, and had a bundle with him tied up in a blue-and-white handkerchief. He went away along the road which would lead him past the spot where the murdered woman was found. He was next seen by two women at about ten minutes after one o'clock, about four hundred yards from the place of the murder. He was running and walking fast, and looking back, and appeared much frightened. He had then no jacket on, and no bundle. At about two o'clock he called at the house of the Rev. George Hadow, at Everleigh, which is about a mile and a quarter from the spot; and he asked to see Mr. Hadow, who went out to him. Mr. Hadow asked him what he wanted. He said, "work." Mr. Hadow said, "I have no work for you; you seem to be a blacksmith." He then observed that the man had no jacket on, was breathing hard, and trembling, as if he had been running very fast, and said to him, "What is the matter with you? What have you been doing?" Upon this, the prisoner immediately hurled two large stones at Mr. Hadow's head. Mr. Hadow rushed in towards him, and succeeded in protecting his head, but received two severe blows on his left arm. Thinking an attempt was being made to murder him, Mr. Hadow ran back into the house, closing the door after him. Recovering the surprise which the suddenness of the attack had caused him, he called his man-servant, and went out, but found that the prisoner was gone. He, however, immediately mounted his horse, and, with his servant, rode in pursuit of him. He very shortly overtook him. The prisoner then said, "I suppose you want me?" Mr. Hadow replied, "Yes; and I am determined to have you." Prisoner then said, "Oh, you are, are you?" and threw three large stones at Mr. Hadow's head with the greatest violence, as fast as possible. The stones struck Mr. Hadow, and wounded him severely. The prisoner then ran away, but two men coming up, he was secured, and taken into custody. One of the men then said to him, "Are you not ashamed to treat a gentleman so in his own house?" The prisoner replied, "No, for I have committed one murder to-day already, and that made me do it!" The man said, "Where?" The prisoner replied, "Oh, you will soon hear of it, and you may as well take me as any one else." He was then given into the custody of a policeman, named Stagg, who was taking him to Devizes, on the charge of assaulting Mr. Hadow, when they were overtaken by another policeman, named Borry, who said the prisoner must be brought back, as a woman had been found dreadfully beaten near Collingbourn. The prisoner then said, "I knocked her down, but I did not ravish her." Borry said, "You ought to be ashamed to tell it." To this, prisoner replied, "I done it. I am tired of living." The other policeman said he wished this had been known before, as it would have saved a journey back. The prisoner then said, "I wonder you did not hear of it before. I met the woman on the road, threw a stone at her, knocked her down, kicked her about the head, and ran away. If I had not gone to the parson's I might have been miles away. If you had not asked me about it, I should have told all about it before we parted." He was then taken to the lock-up house, when he asked if the woman was dead. He was asked, "What woman?" but made no reply. After being committed for trial, when some one spoke of the murder of the woman, the prisoner said, "It was my own tongue that done me." Search was made near the spot of the murder, and a bundle was found which was sworn to as being the one that the prisoner had when he was at the blacksmith's house just before the murder. His jacket was also found on the road, between the spot where the murder was committed and where he was seen by the women coming.

Mr. Smith, for the prisoner, subjected the medical witnesses to a long cross-examination; first, as to the general complexion of homicidal insanity, and the symptoms by which it displayed itself by sudden impulsions to murder, endeavouring to show that the prisoner might have been labouring under that species of mania at the time the murder was committed, and yet appear to be sane afterwards; for, in answer to a question by Mr. Slade, the medical men all said that they considered the prisoner to be perfectly sane.

The case for the prosecution having here closed,

Mr. Smith addressed the jury in a most eloquent and impressive address, contending that the total absence of motive was a strong argument to show that the prisoner must have been insane and a monomaniac at the time he committed the murder, and that it was impossible to account for the act by any other conclusion. He read numerous passages from Dr. Prichard's work on insanity, Dr. Winslow's "Plea of Insanity," and other works. He also called the attention of the jury to the law, as laid down in M'Naughten's case, contending that the prisoner, at the time he committed the act, must have been suffering under that species of mania which exhibits itself in an irresistible inclination to destroy human life, and if the jury believed him to be in such a state of mind as to have no control over his actions, then he was entitled to his acquittal.

Lord Denman, in summing up the case to the jury, observed that the principal question for their consideration, was the state of mind of the prisoner at the time he committed the act which was the cause of the death of the unfortunate woman, for that he was the person who did commit the act was a question almost beyond doubt. They all knew that doctors were in the habit of making theories, but the jury were to say whether those theories were right, and whether there was any proof that the prisoner was under the influence of such morbid affection as to render him irresponsible for his acts. Now, he did not find that a delusion of any kind had been shown. To say a man was irresponsible without positive proof of any act to show that he was labouring under some delusion, seemed to him to be a presumption of knowledge which none but the great Creator himself could possess. He did not himself see any one thing in this case to prove a diseased state of mind in the prisoner, except the violence of the act itself, and he could not help observing that the surgeon of the jail had not found any symptom of disease.

The jury, after a very few minutes' consultation, found the prisoner guilty.

Lord Denman then passed sentence of death upon him in the most impressive manner, not holding out the slightest hope to him that his sentence would be mitigated.

The prisoner, who was a man of most repulsive appearance, walked from the dock with the greatest composure; indeed, he preserved the appearance of the most stolid indifference during the whole trial.—*Western Circuit. Salisbury, March* 12.—*Crown Court. Before Lord Denman.*

OBSERVATIONS.

Our readers will perceive by the report of the last case, that Lord Denman repudiates the idea of insanity, or irresponsibility, in the absence of proof of the presence of a *delusion*. This *dictum* will not bear the test of serious examination. Apart from this, other judges have held the opposite doctrine, admitting the existence of a form of mental derangement, accompanied with a morbid propensity to take away life, unconnected with any delusive impressions or false perceptions. It is time that there should be some approach to uniformity of opinion on this important point among those who are intrusted in this country with the solemn administration of justice.

In France and in America, the judicial tribunals admit a species of insanity, which is termed "homicidal" and "impulsive," in which there exists a motiveless—an overwhelming and uncontrollable desire to take away human life. Lord Denman may deny the fact, and refuse to admit it as a plea in criminal cases, but it does not affect this great psychological truth. God has, in his inscrutable wisdom, willed it, and no legal subtlety can alter the Divine fiat. DELUSION IS NOT THE EXCLUSIVE TEST OF INSANITY: all psychologists admit this,—a man may be mad—may be irresponsible—may be incapable of taking care of himself, or of managing his property, and be free from all delusive ideas! We agree with Lord Denman, that the question of responsibility in reference to crime, all delusion being absent, is a most difficult one; and before the plea is listened to, a strong case of absence of all motive should be conclusively established. We pronounce no opinion on John Smith's case. We do not feel ourselves sufficiently in possession of the facts to enable us to come to a satisfactory judgment.

---

